# Au@Ag Core–Shell Nanoparticles for Colorimetric and Surface-Enhanced Raman-Scattering-Based Multiplex Competitive Lateral Flow Immunoassay for the Simultaneous Detection of Histamine and Parvalbumin in Fish

**DOI:** 10.1021/acsanm.3c04696

**Published:** 2023-12-19

**Authors:** Carlos Fernández-Lodeiro, Lara González-Cabaleiro, Lorena Vázquez-Iglesias, Esther Serrano-Pertierra, Gustavo Bodelón, Mónica Carrera, María Carmen Blanco-López, Jorge Pérez-Juste, Isabel Pastoriza-Santos

**Affiliations:** † CINBIO, Universidade de Vigo, Campus Universitario As Lagoas, Marcosende, 36310 Vigo, Spain; ‡ Department of Physical Chemistry, Universidade de Vigo, Campus Universitario As Lagoas, Marcosende, 36310 Vigo, Spain; § Galicia Sur Health Research Institute (IIS Galicia Sur), 36310 Vigo, Spain; ∥ Department of Biochemistry and Molecular Biology and Institute of Biotechnology of Asturias, University of Oviedo, 33006 Oviedo, Spain; ⊥ Department of Functional Biology and Health Sciences, Universidade de Vigo, 36310 Vigo, Spain; # Department of Food Technology, Spanish National Research Council, Marine Research Institute, 36208 Vigo, Spain; ∇ Department of Physical and Analytical Chemistry and Institute of Biotechnology of Asturias, University of Oviedo, c/Julián Clavería 8, 33006 Oviedo, Spain

**Keywords:** SERS tags, multiplex detection, lateral flow assay, allergens

## Abstract

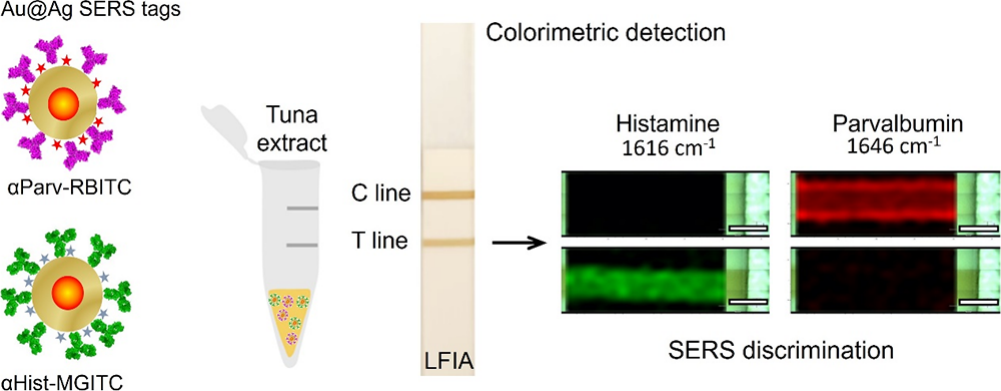

Foodborne allergies and illnesses represent a major global health concern. In particular, fish can trigger life-threatening food allergic reactions and poisoning effects, mainly caused by the ingestion of parvalbumin toxin. Additionally, preformed histamine in less-than-fresh fish serves as a toxicological alert. Consequently, the analytical assessment of parvalbumin and histamine levels in fish becomes a critical public health safety measure. The multiplex detection of both analytes has emerged as an important issue. The analytical detection of parvalbumin and histamine requires different assays; while the determination of parvalbumin is commonly carried out by enzyme-linked immunosorbent assay, histamine is analyzed by high-performance liquid chromatography. In this study, we present an approach for multiplexing detection and quantification of trace amounts of parvalbumin and histamine in canned fish. This is achieved through a colorimetric and surface-enhanced Raman-scattering-based competitive lateral flow assay (SERS-LFIA) employing plasmonic nanoparticles. Two distinct SERS nanotags tailored for histamine or β-parvalbumin detection were synthesized. Initially, spherical 50 nm Au@Ag core–shell nanoparticles (Au@Ag NPs) were encoded with either rhodamine B isothiocyanate (RBITC) or malachite green isothiocyanate (MGITC). Subsequently, these nanoparticles were bioconjugated with anti-β-parvalbumin and antihistamine, forming the basis for our detection and quantification methodology. Additionally, our approach demonstrates the use of SERS-LFIA for the sensitive and multiplexed detection of parvalbumin and histamine on a single test line, paving the way for on-site detection employing portable Raman instruments.

## Introduction

The global demand for fish is steadily increasing worldwide due to its high nutritional value.^1^ Food safety and quality and their associated risks pose a major concern worldwide not only for an economically sustainable food supply chain but also regarding potential danger to consumer health. Thus, awareness of food safety and quality is continuously increasing, resulting in the development of a multidimensional regulatory system that covers all sectors of the food chain, including production, processing, storage, transport, and retail sales.^2^ According to the World Allergy Organization, fish is among the eight major food allergens, which combined are believed to account for more than 90% of worldwide food allergies.^3^ The prevalence of fish allergies in the population ranges from 0.01% in Israel to 7% in Finland.^4^ Allergic reactions to fish are manifested in a variety of symptoms including nausea, vomiting, abdominal pain, dermatitis, asthma, and life-threatening anaphylaxis, even when it is present in small amounts.^5^ Unfortunately, there is no cure for fish allergy, and it can only be managed by the rigorous avoidance of this food and its derivatives in the diet. Parvalbumin is a calcium-binding protein that has been recognized as the major fish allergen, accounting for more than 95% of food allergies associated with fish.^6^ Two isoforms of this protein have been identified; Whereas α-parvalbumins are generally considered nonallergenic, β-parvalbumin is associated with immunoglobulin E (IgE)-mediated food allergic reactions.^7^ Scombroid food poisoning (SFP) is the most common fish-related illness worldwide that develops after consumption of fish containing exogenous histamine generated from bacterial decarboxylation of histidine.^8,9^ The intoxication with this biogenic amine can lead to increased gastric secretion, headache, itching, bronchospasm, and heart arrest if consumed at high concentrations.^10^ Remarkably, the clinical manifestation of histamine intoxication is a pseudoallergic reaction very similar to the IgE-associated food allergy triggered by fish parvalbumin.^11^ The FAO/WHO (Food and Agriculture Organization of the United Nations/World Health Organization) and the European Union have established legislation to set a maximum concentration allowed for histamine in fish and food products of 100 mg kg^–1^ (Commission Regulations (EC) Nos. 2073/2005 and 1019/2013).

The development of rapid, economical, selective, multiplexed, and portable methods for on-site testing has great potential to improve food quality and safety. The established benchmarks for the analytical determination of parvalbumins and histamine in fish and fish products are enzyme-linked immunosorbent assay (ELISA)^12^ and high-performance liquid chromatography,^13^ respectively. The analytical performance of these techniques is unquestionable; nevertheless, these techniques are often limited to the detection of a single analyte per test. Mass spectrometry enabled the simultaneous assessment of multiple analytes. However, its application demands highly trained personnel on bulky and costly instrumentation typically found in centralized laboratories, making it unsuitable for on-site testing. Numerous studies have focused on detecting histamine and parvalbumin in fish, with each study examining these molecules individually^14−17^ However, simultaneous detection of both parvalbumin^18,19^ and histamine^20,21^ can offer a more comprehensive assessment of the safety and freshness of fish for human consumption^22,23^

Colorimetric lateral flow immunoassays (LFIAs) are analytical devices widely used for on-site diagnostics and environmental monitoring^24,25^ that fulfill the WHO’s ASSURED criteria (Affordable, Sensitive, Specific, User-friendly, Rapid and robust, Equipment-free, and Deliverable to end users).^26^ Thus, LFIAs have been widely used in pregnancy tests, infectious disease detection, and drug and food safety testing, as well as for environmental monitoring.^27,28^ For instance, this method has been successfully applied for on-site detection of SARS-CoV-2 during the recent COVID-19 pandemic, owing to its efficacy, simplicity, speed, and cost-effectiveness.^29,30^ Indeed, the U.S. Food and Drug Administration (FDA) has granted emergency use authorization (EUA) to 69 LFIAs. The fundamental principle of the method is the use of plasmonic metal nanoparticles with strong visible light absorption induced by localized surface plasmon resonance phenomena, which allows colorimetric detection with the naked eye. The nanoparticles, previously labeled with antibodies against a given target, move by capillary action along a strip until being captured in the test (T) and control (C) lines generally by immobilized antibodies, generally. Even though the LFIA can offer rapid and qualitative results, the use of colorimetric detection significantly affects its sensitivity and multiplexing capabilities.^31−33^ Additionally, the LFIA can be versatilely configured into different assay formats, including competitive and sandwich assays. While the sandwich assay is more suitable for high-molecular-weight (MW) analytes with multiple epitopes, the competitive assay is preferable for low-MW target analytes (single epitope). A positive outcome in a competitive assay is characterized by the absence of color in the T line, indicating the hindrance of antibodies’ interaction with immobilized receptors by target analytes. Conversely, negative results are represented by intensities in both the T and C lines.^31−33^

A powerful means to overcome the limitations of colorimetric LFIA is to combine this analytical method with surface-enhanced Raman scattering (SERS) spectroscopy.^34−36^ Moreover, the existence of hand-held Raman instruments allows on-site testing.^37,38^ SERS-based LFIA is an emerging analytical method that has been recently developed for the detection and quantification of viruses, bacteria, toxins, and contaminants.^39−42^ This modality of detection makes use of the so-called SERS tags, which are composed of a plasmonic metal nanoparticle encoded with a Raman reporter and functionalized with a targeting entity (e.g., antibodies, aptamers). The SERS nanoprobes feature several benefits over fluorescent and colorimetric optical labels, such as higher photostability, signal intensity, and multiplexing capabilities, as well as the capacity to use a single laser line for excitation in multiplexed detection formats.^43^

**Scheme 1:**
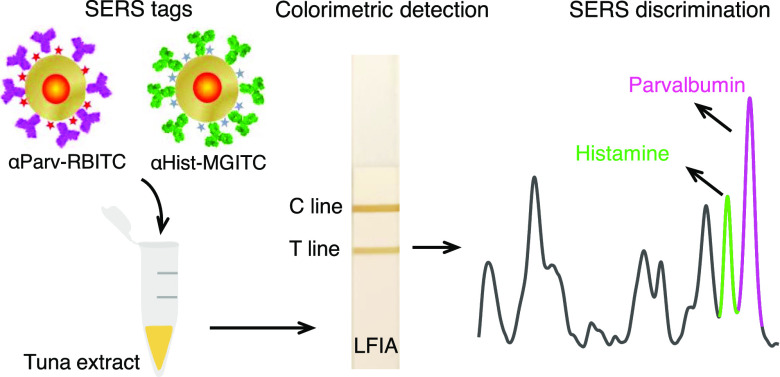
Schematic Depicting a Dual Colorimetric and SERS-Based Competitive LFIA for Simultaneous Detection and Quantification of Parvalbumin and Histamine Two well-differentiated SERS tags conjugated with anti-β-parvalbumin (αParv) and anti-histamine (αHist) are synthesized and mixed with a canned tuna extract. Subsequently, a dual colorimetric SERS-based detection and discrimination of the antigens is performed.

In this work, we aim to develop a colorimetric and SERS-based competitive LFIA for simultaneous detection and discrimination of parvalbumin and histamine in canned fish in a single T line (see Scheme 1). As nanoprobes, we design 50 nm Au@Ag core–shell nanoparticles (Au@Ag NPs) codified with either rhodamine B isothiocyanate (RBITC) or malachite green isothiocyanate (MGITC) and bioconjugated with anti-β-parvalbumin and antihistamine. We investigate the optimal LFIA conditions to avoid nonspecific interactions and cross-reactivity. As a proof-of-concept, we evaluate the method in canned tuna as the food matrix.^44−46^ Tuna, belonging to the *Scombroidae* family, is characterized by high levels of histidine, which might be transformed to histamine throughout the food chain and canning process.^44,47^ Remarkably, histamine presents high thermal stability, and therefore it might withstand food processing for canning.^48^ The amount of parvalbumin differs considerably among different fish species and tissues.^49^ In tuna it is significantly higher in white than in red muscle, as well as in ventral and dorsal portions of the white muscle.^50^ Likewise histamine, parvalbumin is also thermally stable, and its content in canned food might vary depending on techniques employed during food processing.^51,52^ Therefore, the development of strategies for the detection and quantification of histamine and parvalbumin in canned tuna fish is of relevance.^52,53^

## Results and Discussion

### Synthesis and Analysis of SERS Tags

**Figure 1:**
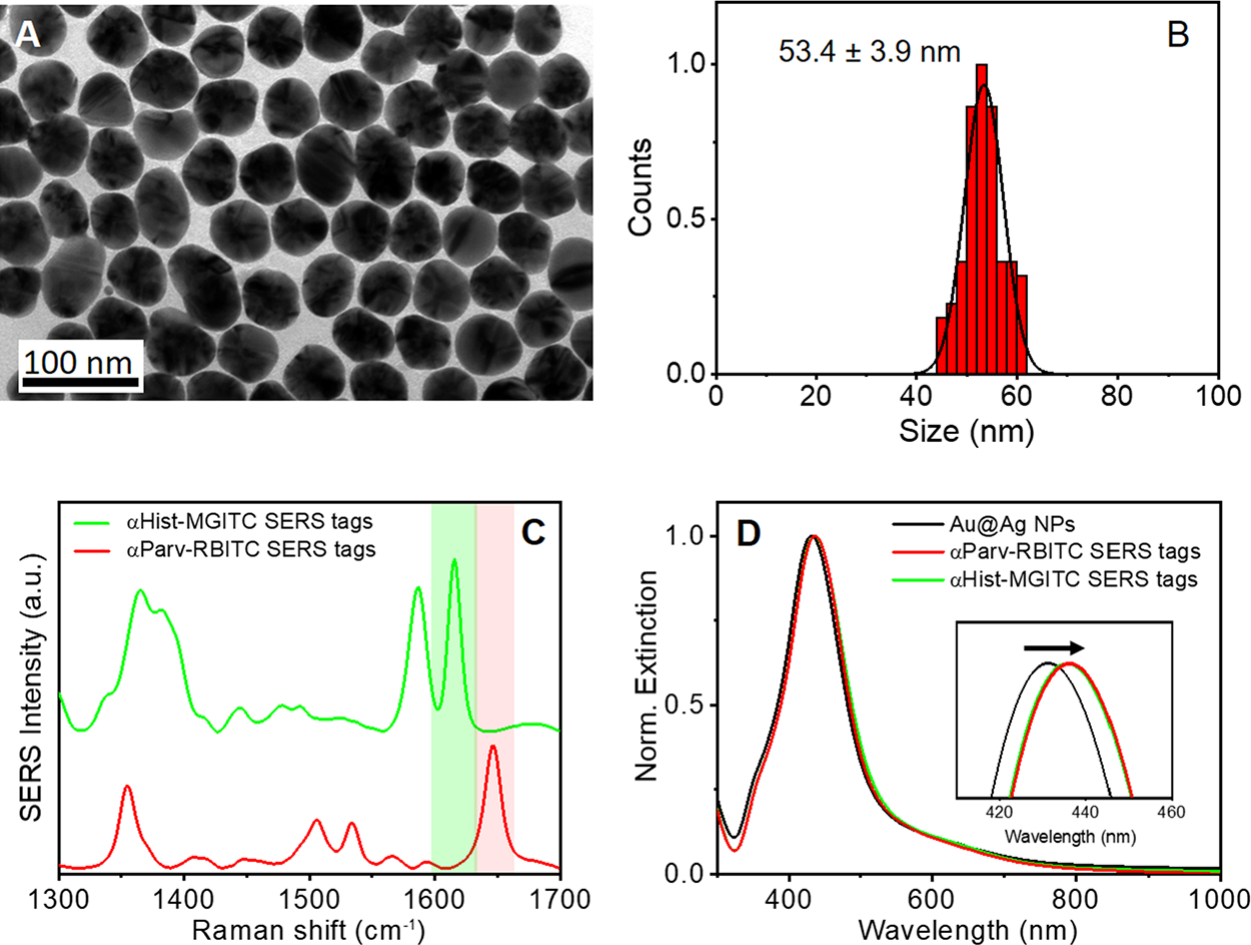
SERS tag characterization. (A) Representative TEM image of spherical Au@Ag core–shell nanoparticles (Au@Ag NPs). (B) Size distribution histogram of Au@Ag NPs. (C) SERS spectra of the SERS tags encoded with MGITC (green) and RBITC (red). The green and red shadowed regions indicate 1616 and 1646 cm^–1^ Raman peaks of MGITC and RBITC-encoded SERS tags, respectively. (D) Normalized extinction spectra of Au@Ag NPs before (black) and after functionalization with RBITC and anti-β-parvalbumin (red line) and with MGITC and antihistamine (green line). The inset clearly shows the red shift in the plasmon band peak after the codification of Au@Ag NPs.

For the fabrication of the SERS tags specific for histamine or β-parvalbumin, we chose spherical Au@Ag core–shell nanoparticles (Au@Ag NPs) as plasmonic nanoparticles since they exhibit stronger extinction cross-section and SERS efficiency than Au nanospheres.^54^ Thus, uniform citrate-stabilized Au@Ag NPs of ca. 54 nm (Figure 1A, B) were synthesized by a seed-mediated growth approach employing iron(II) as a reducing agent at room temperature.^55^ The Au@Ag NPs exhibited a localized surface plasmon band at 430 nm (Figure 1C). Remarkably, the use of citrate as a capping ligand facilitates its further surface modification with thiolated molecules and proteins.^55^ For the nanoparticle codification with Raman reporters, we employed rhodamine B isothiocyanate (RBITC) and malachite green isothiocyanate (MGITC), as both molecules present high Raman cross-section and characteristic Raman peaks that readily allow their differentiation in mixtures by SERS (1616 cm^–1^ assigned to aromatic C–C stretching for MGITC and 1646 cm^–1^ assigned to C–C stretching of the xanthene ring for RBITC, Figure 1C). A full vibrational assignment of the SERS spectra of RBITC or MGITC can be found in Figures S1 and S2. Au@Ag NPs were codified with either RBITC or MGITC via ligand exchange (see the Experimental Section for further details). Finally, the Raman-codified Au@Ag NPs were conjugated with monoclonal antibodies against histamine or β-parvalbumin through physical adsorption.^54^ More precisely, SERS tags encoded with RBITC were functionalized with anti-β-parvalbumin (αParv-RBITC SERS tags) and MGITC-encoded ones with antihistamine (αHist-MGITC SERS tag). As expected, the surface modification of Au@Ag NPs with Raman reports and antibodies produced a slight red shift in the localized surface plasmon resonance due to changes in the local refractive index (Figure 1D).

For multiplexed LFIA detection, the composition of the running buffer is key to reducing the nonspecific binding and cross-reactivity between the SERS tags and the immobilized antigens in the T lines. It should be noted that we immobilized parvalbumin and histamine hapten in two T lines, T Parv and T Hist (Figures S4A and S3B) since it was intended to follow a competitive strategy for the detection of parvalbumin and histamine. In this work, we assessed by colorimetric LFIA two different running buffers: borate buffer (BB), and phosphate buffer (PB) at two pHs, 7.4 and 8.4. Whereas BB triggered nonspecific binding between αHist-MGITC SERS tags and immobilized parvalbumin (Figure S3A, strip 2), and αParv-RBITC SERS tags with immobilized histamine (Figure S3B, strips 1 and 2), the use of PB for the detection of histamine or parvalbumin resulted in no apparent cross-reactivity whatsoever (Figure S3A,B, strips 3 and 4). Since the intensity of the signals in PB was not influenced by the pHs assessed, we selected PB at pH 7.4 as the running buffer. It is important to note that the colorimetric signal observed in the C line corresponds to both SERS tags bound to the immobilized protein-G.

Surfactants, such as Tween 20, are commonly used in LFIA to improve the flow of the sample and reagents through the nitrocellulose membrane. Therefore, we assessed two different concentrations of Tween 20 (1 and 3% w/w) in PB (pH 7.4), for the optical detection of histamine and parvalbumin immobilized in the LFIA strip. Quantification of the colorimetric signal in the T and C lines was carried out by ImageJ software (see the Experimental section). As observed in parts C and S3D for histamine and parvalbumin, respectively, the highest surfactant concentration leads to higher color intensities. Therefore, Tween 20 at 3% w/w was included in the PB running buffer. Next, we studied the use of BSA or casein (1% w/w) as blocking agents in PB pH 7.4 and Tween 20 (3% w/w) to avoid/reduce nonspecific binding, thereby increasing the specificity and sensitivity of the LFIA. As observed in Figure S3C,D for histamine and parvalbumin, respectively, the use of casein as a blocking agent leads to higher color intensities. Therefore, Tween 20 (3% w/w) and casein (1% w/w) were selected as components of the PB running buffer at pH 7.4.

**Figure 2:**
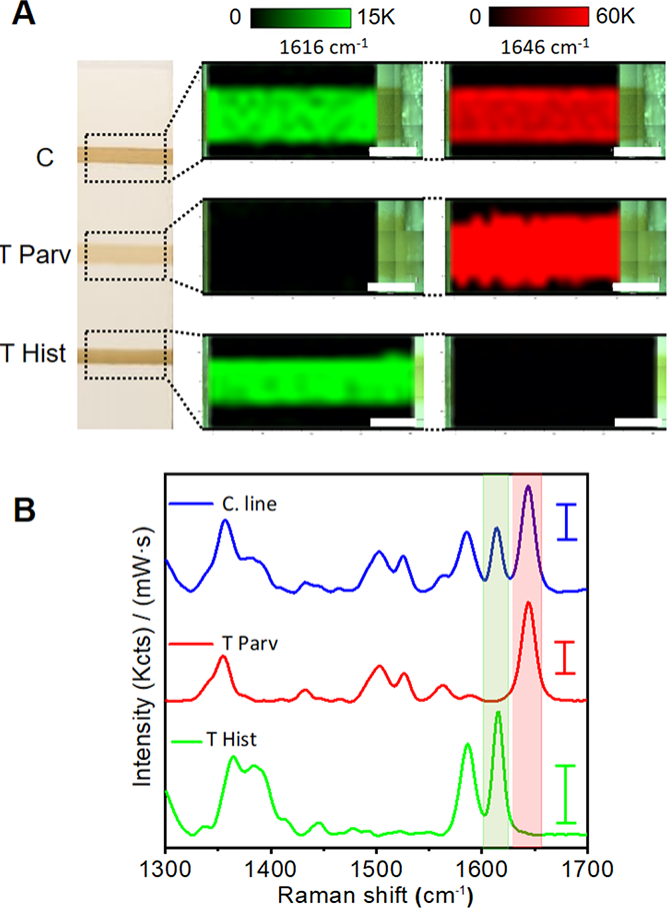
Nanoprobe cross-reactivity assessment. (A) Photograph of the LFIA strip with histamine (Hist), parvalbumin (Parv), and protein-G (PG) immobilized in the test (T) and control (C) lines, as indicated. SERS intensity mappings acquired at 1616 and 1646 cm^–1^ which are characteristic peaks of αHist-MGITC SERS or αParv-RBITC SERS tags, respectively. (B) Average SERS spectra from the 20 highest intensity points measured in each line. The green and red shadowed regions indicate 1616 and 1646 cm^–1^ Raman peaks of the MGITC and RBITC-encoded tags. Scale bars in (A) represent 1 mm. Scale bars in (B) represent 1 Kcts mW^–1^ s^–1^. All SERS measurements were carried out with a 532 nm laser line, 10× objective, 0.25 mW laser power, acquisition time 0.5 s, and 231 points.

Next, we assessed the potential cross-reactivity of the SERS tags. The colorimetric readout of the LFIA strips demonstrates the absence of cross-reactivity between the SERS tags and their targets when used individually in the assay (Figure S4A,B, strips 3 in both cases). Thus, no binding of αParv-RBITC SERS tags and αHist- MGITC SERS tags is observed in the histamine and parvalbumin immobilized T lines, respectively. Finally, we investigated the antigen binding specificity of the two nanoprobes when used simultaneously in the LFIA and having each target immobilized in a different T line, as shown in Figure 2A. To assess it, we cannot use the colorimetric LFIA since both nanoprobes exhibit similar extinction features (Figure 1C) but SERS LFIA. Figure 2A shows the SERS intensity mappings acquired at 1616 and 1646 cm^–1^ (characteristic Raman peaks for αHist-MGITC and αParv-RBITC SERS tags, respectively) in the C and T lines. The results show that SERS tags bind specifically to their cognate antigens, eliciting a homogeneous distribution of the recorded SERS signal along the lines. Thus, no signal of αParv-RBITC SERS is observed in the Hist immobilized line, and the same happens with the αHist-MGITC SERS tags in the Parv immobilized line. The absence of any cross-reactivity between the SERS tags in the T lines is also evidenced in the representative average SERS spectra from both T lines shown in Figure 2B. As expected, both SERS tags are detected in the C line (protein G) with highly homogeneous signals (Figure 2A), which is also evidenced in the representative average SERS spectra (blue spectrum, Figure 2B). These results demonstrate the selectivity and absence of cross-reactivity of the proposed SERS-based LFIA approach for histamine and parvalbumin detection.

#### Development of the Competitive SERS-Based LFIA for Detection of Histamine and Parvalbumin

**Figure 3:**
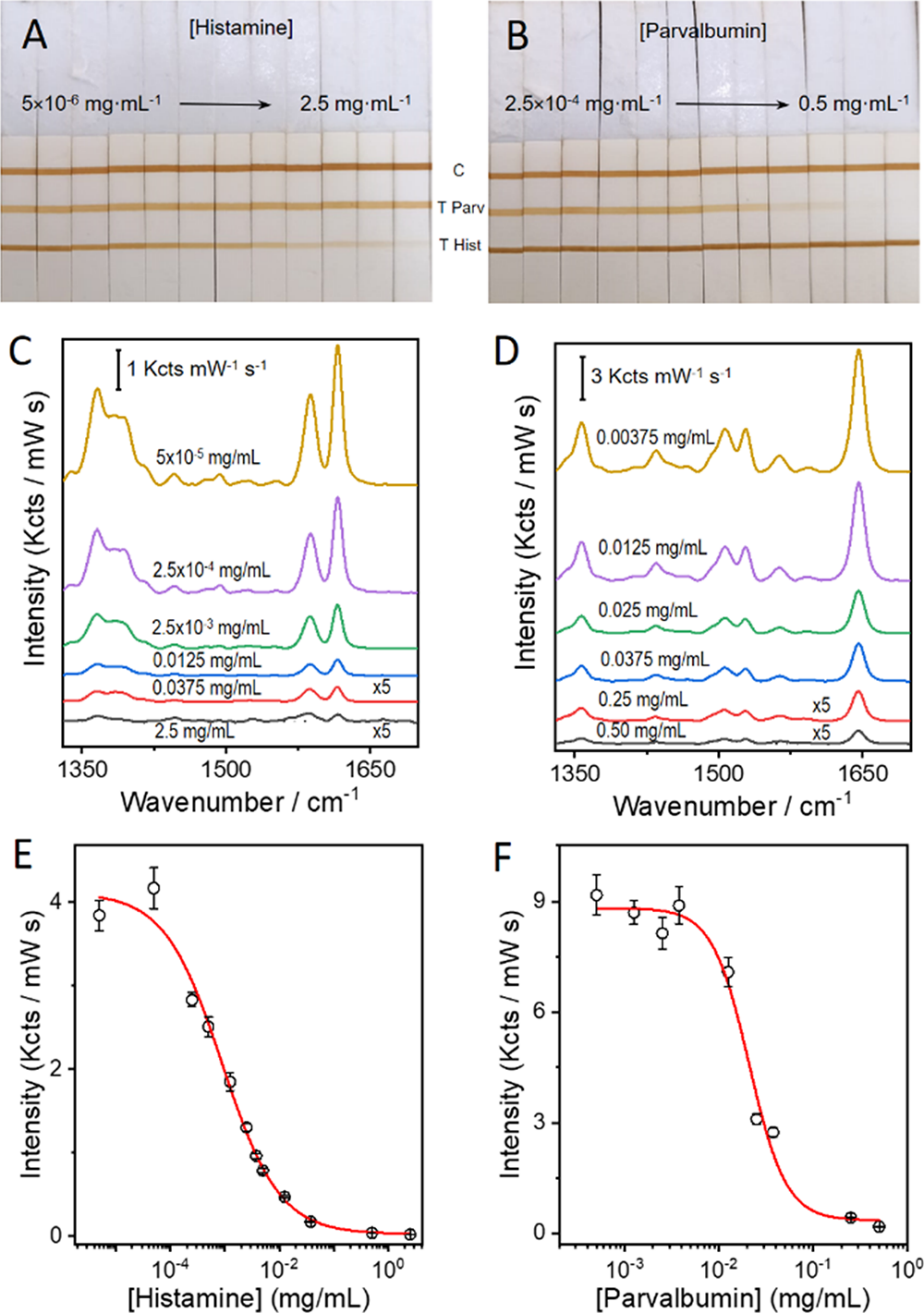
(A and B) Photographs of LFIA strips after running αParv-RBITC SERS tags and αHist- MGITC SERS tags previously incubated with different concentrations of (A) histamine (from 2.5 to 5 × 10^–6^ mg mL^–1^) or (B) parvalbumin (from 0.5 to 2.5 × 10^–4^ mg mL^–1^) in PB. (C and D) Average SERS spectra acquired from the different Hist (C) and Parv (D) T lines are shown in (A) and (B), respectively. (E, F) Variation of SERS intensity at 1646 cm^–1^ (E) or 1616 cm^–1^ (F) with the concentration of parvalbumin and histamine, respectively. The red lines represent the fitting of the SERS intensity measurements to a four-parameter sigmoid equation. Standard deviations correspond to the 20 higher-intensity SERS points of each strip. All SERS measurements were carried out with a 532 nm laser line, 10× objective, 0.25, 2.31, or 12.50 mW laser power depending on the color intensity of the test lines, 1.0 s acquisition time, and 143 points.

Since it is a competitive assay, the free target analytes (i.e., histamine/parvalbumin) present in the sample are expected to compete with the immobilized antigens in the T lines for binding with their respective SERS tags. Thus, the lower the histamine/parvalbumin concentration in the sample, the higher the signal in the T lines. Conversely, the higher the histamine/parvalbumin concentration in the sample, the lower the signal in the T lines. It was proved using colorimetric LFIA by incubating simultaneously αHist-MGITC SERS tags and αParv-RBITC SERS tags with different concentrations of histamine (from 5 × 10^–6^ to 2.5 mg mL^–1^) or parvalbumin (from 2.5 × 10^–4^ to 0.5 mg mL^–1^) in PB (see Methods section). As seen in Figure 3, the colorimetric signal in the histamine or parvalbumin T lines increases with a decreasing concentration of free histamine (Figure 3A) or parvalbumin (Figure 3B), respectively. Importantly, the colorimetric signal corresponding to parvalbumin (Figure 3B) or histamine (Figure 3A) in the T lines remains constant, demonstrating the specificity of the assay. Besides, it should be noted that regardless of the target concentration, the C lines show a constant color intensity, confirming the reliability of the method. These two experiments were employed to obtain calibration SERS curves for both antigens. Thus, the SERS spectra were acquired in the histidine (Figure 3C) and parvalbumin (Figure 3D) T lines for experiments performed with different target concentrations. As shown in Figure 3C, D, the intensity of the SERS signals decreases when the target concentration. Figure 3E, F plot the SERS intensity at 1616 cm^–1^ (Hist) and 1646 cm^–1^ (Parv), respectively, as a function of the antigen concentration, and in both cases, the data fit a sigmoid-shaped profile. The equation employed was the four-parameter logistic (4PL) equation which is commonly used in competitive immunoassays.^56^ When the antigen concentration is too low, the curve presents an asymptotic behavior due to the saturation of the immobilized antigen of the T line by the SERS tags. On the other hand, when the antigen concentration is too high, the curve also presents an asymptotic behavior since no signal is presented. The 4PL equation is represented by
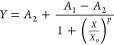
1where *Y* is the sensor measurement and *X* is the antigen concentration. *A*_1_ and *A*_2_ are the *S*-values of the upper and lower asymptote, respectively, *p* is the slope at the inflection point and *X*_0_ corresponds to the value of *X* corresponding to 50% of the maximum asymptote.^57^Table 1 summarizes the values obtained from the fitting of the SERS measurements to a 4PL equation for the histamine and parvalbumin.

**Table 1:** Four-Parameter Logistic Equation Values Obtained from the Calibration Curves of Histamine and Parvalbumin Using the SERS Detection Method

antigen	*A*_1_	*A*_2_	*X*_0_ (mg mL^–1^)	*p*	*R*^2^	IC_10_/LOD (mg mL^–1^)	IC_20_ (mg mL^–1^)	IC_80_ (mg mL^–1^)
parvalbumin	8822.3 ± 297.4	358.3 ± 391.2	0.021 ± 0.002	2.22 ± 0.50	0.979	7.74 × 10^–3^	1.12 × 10^–2^	3.90 × 10^–2^
histamine	4113.9 ± 182.6	23.2 ± 120.0	(8.9 ± 1.6)×10^–4^	0.83 ± 0.11	0.984	6.29 × 10^–5^	1.67 × 10^–4^	4.73 × 10^–3^

The limits of detection (LOD), determined as the concentration of antigen that generates 10% of the signal of the control samples (IC10), were 6.29 × 10^–5^ and 7.74 × 10^–3^ mg mL^–1^ for histamine and parvalbumin, respectively. To establish the quantification range, the 20–80% inhibition (IC_20_–IC_80_) criteria were used.^58,59^ For parvalbumin, the quantification range was 0.0112 and 0.039 mg mL^–1^, while for histamine, it was 1.67 × 10^–4^ and 4.73 × 10^–3^ mg mL^–1^.

A similar analysis was performed with an optical reader. Figure S5 shows the colorimetric calibration curves for parvalbumin and histamine and Table S1 summarizes the values obtained from the fitting to a 4PL equation. It should be noted that the LODs and quantification ranges obtained were similar to those determined by SERS.

#### Quantitative Detection of Spiked Histamine and Parvalbumin in Canned Tuna by a Dual Colorimetric SERS-LFIA

**Figure 4:**
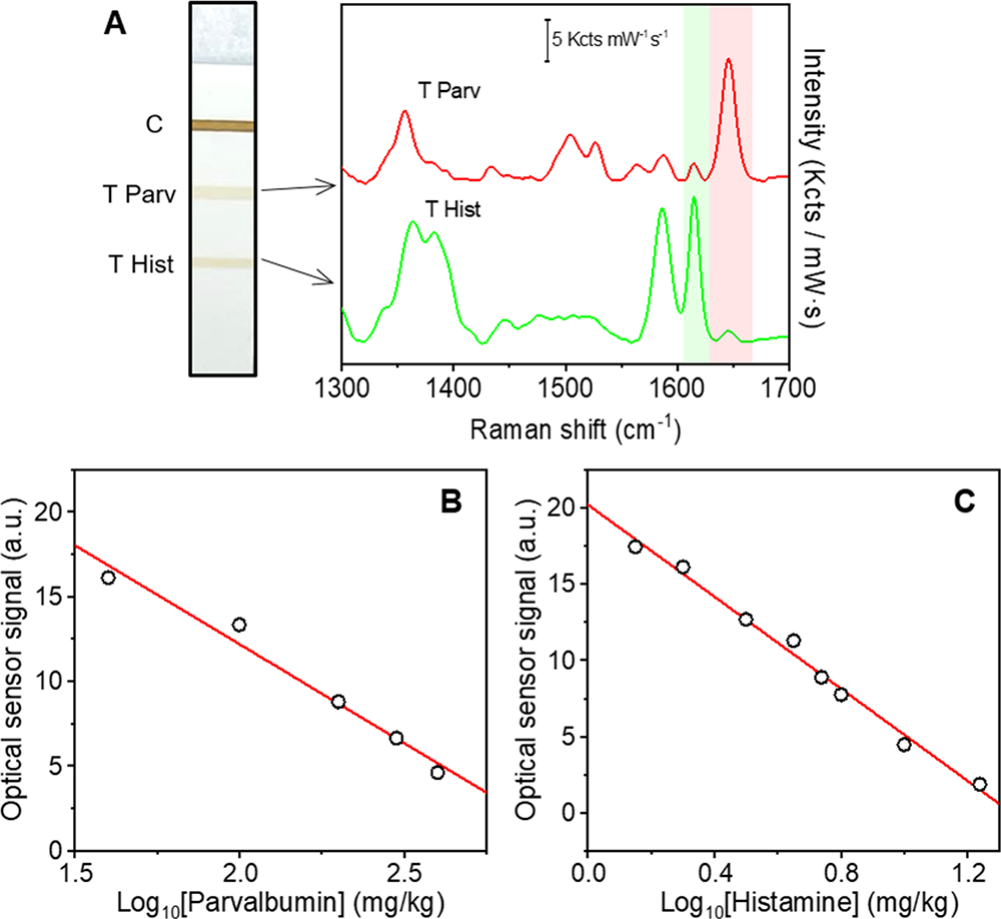
(A) Photograph of an LFIA strip with a control (C) line and two test lines for parvalbumin (T Parv) and histamine (T Hist), as indicated, and representative SERS spectra measured in each T line of the LFIA strip with a hand-held Raman spectrometer with a 532 nm laser line, 21 mW laser power, and 1.0 s acquisition time. The scale bar represents 5 Kcts mW^–1^ s^–1^. (B, C) Optical sensor linear regression range of parvalbumin (B) and histamine (C) obtained by analyzing extracts of canned tuna.

To emulate a positive sample for histamine and parvalbumin in canned tuna fish, 2 g of dried canned tuna were extracted as reported previously;^60^ the extract was diluted 10-fold in PBS 1× to reduce matrix effects and spiked with different amounts of histamine or/and parvalbumin. Before, the colorimetric LFIA quantification of the samples, we investigated by SERS the specificity of the αParv-RBITC and αHist-MGITC SERS tags in this complex matrix by running an extract containing both nanoprobes and histamine and parvalbumin. As shown in Figure 4A, SERS analysis of the histamine and parvalbumin T lines (printed in the same strip) with a portable spectrophotometer demonstrated the specificity of the αParv-RBITC SERS tags and αHist-MGITC SERS nanoprobes. The spectra recorded in each T line exhibit the characteristic Raman peaks of either αParv-RBITC SERS tags (red spectrum, Figure 4A) or αHist-MGITC SERS tags (green spectrum, Figure 4A).

Next, we performed colorimetric LFIA quantification of spiked histamine and parvalbumin in canned tuna extract. As shown in Figure S5, as the concentration of histamine or parvalbumin increases the color intensity of the corresponding T line decreases. The analysis of the optical signal from the LFIA strips allowed us to obtain the calibration curves for both antigens (Figure S5C,D). The quantification range (3–120 mg/kg for histamine (Figure 4B) and 94–597 mg/kg for parvalbumin (Figure 4C) was established by the IC_20_-IC_80_ criteria^58,59^ and nicely fit with the calibration line and showed no rejection of outliers. Besides, parvalbumin and histamine concentrations in mg/kg can be expressed quantitatively as a function of the Optical Sensor signal (O.S.) by empirical formulas: log*[Parvalbumin]* = −11.7 × O.S. + 35.5 (*R*^2^ = 0.97) and log*[Histamine]* = −7.5 × O.S. + 20.2 (*R*^2^ = 0.99). A similar analysis was performed via SERS measurements. Figure S6 shows the SERS-based calibration curves for parvalbumin and histamine. Table S3 summarizes the values obtained from the fitting to the 4PL equation. It should be noted that the LODs and the quantification ranges obtained were similar to those determined by an optical reader.

Considering that the European Union adopted a histamine limit of 100 mg/kg in canned products and the U.S. of 50 mg/kg,^61^ these values are within the calibration range of our sensor which has an LOD of 1 mg/kg for histamine (Table S2). Therefore, the developed sensor is ideal for quantifying the levels of histamine. In the case of parvalbumin, the calculated LOD was 33.4 mg/kg (Table S2). Although there are no legal limits for parvalbumin, its content is directly correlated with the allergenicity of fish^49^ Hence, its quantification is important for risk assessment and to aid consumers in deciding whether it can trigger an allergic reaction. Subsequently, once the calibration curves and LOD were determined, we checked the accuracy of the sensor by estimating the recovery of histamine and parvalbumin in a set of spiked samples. The recovery was determined by interpolating the color intensity obtained from the tuna extract spike experiment on the calibration curve to derive the concentration of the allergen, taking into account the dilutions made (see the Experimental Section for further details). As shown in Table 2, the recoveries range from 90 to 110% for both antigens. Therefore, we can conclude that the sensor may be employed to detect and quantify histamine and parvalbumin in canned tuna fish by the combination of optical readout and SERS.

**Table 2:** Accuracy of the Colorimetric LFIA Sensor for Histamine and Parvalbumin Detection in Tuna Fish Spiked Samples

	spiked (mg/kg)	found (mg/kg)	recovery(%)
histamine	1	0.85	85.0
	5	4.6	92.6
	10	9.8	98.5
	15	13.8	92.3
	20	22	109.5
	50	50	100.4
parvalbumin	20	28	121.4
	50	48	111.8
	100	88	109.8
	150	170	111.0
	200	192	88.4

#### Multiplexed SERS Detection in a Single Test Line of Spiked Histamine and Parvalbumin in Canned Tuna

**Figure 5:**
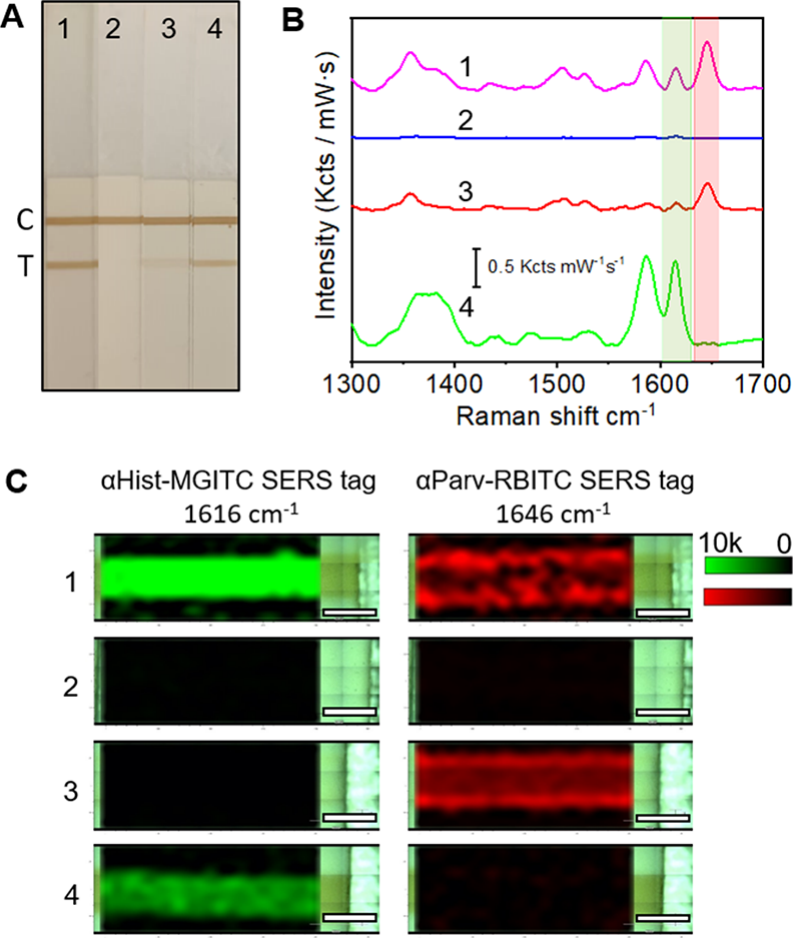
(A) Photograph of four LFIA strips corresponding to experiments performed in the absence of histamine and parvalbumin (1), the presence of histamine and parvalbumin in excess (2), the presence of parvalbumin and no histamine (3) and the presence of histamine and no parvalbumin (4) immobilized in the test (T) line. (B) Representative SERS spectra measured with a hand-held Raman in the different T lines, as indicated. SERS measurements were performed with a 532 nm laser line, 21 mW and 1 s acquisition time. The shadowed regions indicate characteristic Raman peaks of αHist-MGITC SERS tags (1616 cm^–1^, green) and αParv-RBITC SERS tags (1646 cm^–1^, in red). The scale bar represents 0.5 Kcts mW^–1^ s^–1^. (C) SERS intensity mappings acquired at 1616 cm^–1^ (left) and 1646 cm^–1^ (right) in the different T lines from (A), as indicated, showing the presence/absence and spatial distribution of αParv-RBITC SERS tags and αHist-MGITC SERS tags, respectively. Scale bars are 1 mm. SERS mappings were carried out with a 532 nm laser line, 10× objective, 2.31 or 12.50 mW laser power depending on the color intensity of the test lines, acquisition time 1.0 s, and 231 points.

The fingerprinting feature of SERS opens the possibility of developing a competitive SERS-based LFIA for the simultaneous detection of both antigens in a single T line. To assess this, parvalbumin and the histamine hapten (histamine-BSA conjugate) were mixed and immobilized in a single T line. In addition, before the lateral flow assay, the αHist-MGITC and αParv-RBITC SERS tags were incubated in canned tuna extract diluted in PB with no antigens (sample 1), with an excess of both antigens (20 g/kg histamine and 50 g/kg parvalbumin, sample 2), or with just one antigen in excess (20 g/kg histamine and no parvalbumin in sample 3 and 50 g/kg parvalbumin and no histamine in sample 4). An excess of antigens means an amount that is enough to saturate the nanoprobe binding sites. As expected, the analysis of the colorimetric output of the T lines in the LFIAs (Figure 5A) shows a colored band in the absence of antigens (sample 1, strip 1), and no signal when the SERS tags were incubated with both antigens in excess (sample 2, strip 2). The signal, although less intense, is also evident in the T line upon incubation of the SERS tags with either histamine (sample 3, strip 3) or parvalbumin (sample 4, strip 4). Hence, the colorimetric assay is not unsuitable for single T-line strips. Using a portable Raman instrument, we analyzed the T lines by SERS demonstrating that in the absence of the two antigens (strip 1), both SERS tags bound to the antigens immobilized in the T line. Thus, the SERS spectra recorded in the strip showed the characteristic Raman peaks from αParv-RBITC SERS tags and αHist-MGITC SERS tags (Figure 5B). It is also evidenced in the SERS intensity mappings acquired at 1616 cm^–1^ (Figure 5C, left) and 1646 cm^–1^ (Figure 5C, right), which show the spatial distribution of αHist-MGITC SERS tags and αParv-RBITC SERS tags in the T line. On the contrary, when SERS tags were incubated with antigens in excess, no SERS signals were detected in the T line (strip 2, Figure 5B, C). Finally, when incubated with only one of the two antigens, the SERS signal detected in the T line corresponds to the opposite SERS tag (samples 2 and 3, Figure 5B, C). Interestingly, no cross-reactivity was observed in any case. It should be noted that using the colorimetric approach, only sample 2 containing an excess of both antigens (colorless T line) could be reliably evaluated. Thus, the proposed competitive SERS-based LFIA allowed for the detection of histamine and parvalbumin in a single T line, paving the way for the rapid multiplex detection of fish antigens and allergens in the same sample.

## Conclusions

A biosensor for the multiplex detection of parvalbumin and histamine has been developed based on the combination of a competitive colorimetric lateral flow immunoassay and SERS spectroscopy. The proposed method is based on two identical Au@Ag SERS tags encoded with two different Raman reporters: RBITC and MGITC. Each nanoprobe bioconjugated with monoclonal antibodies against histamine or parvalbumin enabled the specific detection of both antigens with no cross-reactivity. The simplicity and specificity of the LFIA technique combined with the high sensitivity of SERS spectroscopy allowed for the detection and quantification of both antigens. Colorimetric assays offer quicker readings and enable quantification, but when SERS spectroscopy is used, it becomes feasible to detect and differentiate between two allergens within the same test line. Conversely, with optical readers, while it is possible to determine if the sample contains allergens or not, it lacks the capability to discriminate between them. The SERS LODs (IC_10_) obtained for canned tuna extract were 1.0 and 33.4 mg/kg for histamine and parvalbumin, respectively. Furthermore, the quantification ranges estimated from (IC_20_–IC_80_) were from 3 to 120 mg/kg and from 94 to 597 mg/kg for histamine and parvalbumin, respectively. Considering that the legal histamine concentration in tuna fish by the European Union is 50 mg/kg, the sensor meets a successful range of quantification. In addition, the multiplexing capabilities of SERS allowed the detection of both antigens in the same T-line strip, which paved the way for the development of LFIA with highly multiplexing capabilities.

## Experimental Section

### Materials

Mouse histamine monoclonal antibody (MBS2025715) and histamine-BSA conjugate antigen (MBS358205) were purchased from Mybiosource. Protein G was purchased from GenScript. β-parvalbumin monoclonal antibody (PV235 PUR) was purchased from Swant. Bovine serum albumin (BSA, ≥ 98%), casein sodium salt from bovine milk, sucrose (99.5%), sodium phosphate monobasic (≥98%), Tween 20, boric acid (99.5%), iron(II) sulfate heptahydrate (≥99%), silver nitrate (≥99%), sodium citrate tribasic dihydrate (≥98%), ethylenediaminetetraacetic acid tetrasodium salt hydrate (EDTA, 99%), rhodamine B isothiocyanate (RBIT), and phosphate-buffered saline (PBS 10×) were purchased from Sigma-Aldrich. Hydrogen tetrachloroaurate (III) trihydrate (99.99%) was supplied by Alfa Aesar. Sulfuric acid (95–97%) was supplied by Scharlau. Citric acid monohydrate (99.5%) and sodium phosphate dibasic acid (≥99%) were obtained from Fluka. Malachite green isothiocyanate (MGITC) was purchased from Invitrogen. Nitrocellulose membranes (UniSart CN95) were purchased from Sartorius. Absorbent pads (CF6) and backing cards (10547158) were purchased from Cytiva. Parvalbumin antigen was isolated at the Marine Research Institute (IIM), CSIC, Vigo. All chemicals were used as received, and ultrapure water (type I) was used in all the preparations.

### Instrumentation

IsoFlow reagent dispensing system (Imagene Techology, USA) was used to dispense the control and test lines. A guillotine Fellows Gamma instrument was used to cut the strips.

SERS experiments were conducted with a Renishaw InVia Reflex confocal system. The spectrograph used a high-resolution grating (1800 grooves per millimeter) with additional band-pass filter optics, a confocal microscope, and a 2D-CCD camera. SERS mappings were obtained using a point-mapping method with a 10× objective (N.A. 0.25), which provided a spatial resolution of about 5.3 μm.^2^ It created a spectral image by measuring the SERS spectrum of each pixel of the image one at a time. Laser excitation was carried out at 532 nm with 12.50, 2.31, and 0.255 mW of power and a 1 s acquisition time. All of the SERS measurements were normalized by laser power and acquisition time. The SERS images of each well were decoded using the characteristic peak of the Raman reporter molecule (rhodamine B isothiocyanate (RBITC), 1646 cm^–1^ and malachite green isothiocyanate (MGITC) 1616 cm^–1^) using WiRE software V 4.1 (Renishaw, UK).

To characterize the optical density of control and test lines, a ChemiDocTM XRS+ was used to obtain photographs of the strips. After the acquisition, they were analyzed employing the ImageJ 1.49v software.

Optical characterization of the colloids was carried out using a Cary 300 UV–vis spectrophotometer (Varian, Salt Lake City, UT, USA). TEM images were acquired with a JEOL JEM 1010 TEM instrument operating at an acceleration voltage of 100 kV.

### Methods

#### Synthesis of Citrate-Stabilized Au@Ag NPs

The synthesis is a seeded growth methodology reported by Fernández-Lodeiro et al.^55^

##### Synthesis of 14.0 nm Au Seeds

Small citrate-stabilized Au NPs were prepared following the method previously reported by Schulz et al.^62^ Briefly, 150 mL of 2.2 mM citrate buffer (75/25 sodium citrate/citric acid) was heated in a three-neck round-bottom flask to its boiling point. After 15 min, EDTA was added to reach a molar concentration of 0.01 mM. Subsequently, 1 mL of HAuCl_4_ 25 mM was added. It was allowed to react for 10 min until a red wine color was achieved, and then the colloid was cooled until room temperature.

##### Synthesis of Au@Ag Core–Shell NPs

First, 10 mL of 14.0 nm Au seeds (0.15 mM in Au^0^) were mixed with 0.3 mL of sodium citrate 100 mM, 30 μL of 1 M H_2_SO_4_, and 4.67 mL of ultrapure water. The final pH was 4.0. The Au seed growth was performed in multiple steps. In the first overgrowth, at this Au seeds solution, 15 mL of AgNO_3_ 1 mM and 15 mL of reducing solution containing 4 mM FeSO_4_ and 4 mM sodium citrate were simultaneously added using a double syringe pump at 90 mL/hour. After finishing the addition of the reactants (10 min) the Ag growth is complete. Finally, 0.9 mL of 100 mM sodium citrate was added to improve the colloidal stability. In a second overgrowth step, the protocol is the same as for the first overgrowth but using as seeds 15.3 mL of Au@Ag colloid obtained in the previous overgrowth step. The final nanoparticle size was 53 nm. The 45.3 mL of colloid were centrifuged (1160 *g* × 30 min). The pellet was resuspended in 4.5 mL of 1 mM sodium citrate 1 mM.

#### Fabrication of SERS-Encoded NPs

To codify the Au@Ag NPs, 100 μL of the concentrated colloid was diluted in 675 μL of ultrapure water. After the dilution, the codification with the Raman probes was carried out by adding 100 μL of a solution of rhodamine B isothiocyanate (RBITC) (10 × 10^–7^ M) or 15 μL of malachite green isothiocyanate (MGITC) (10 × 10^–6^ M) in ethanol mixed with vortex and kept undisturbed for 30 min. After 30 min, 750 μL of borate buffer 10 mM and pH = 8.5 was added in the case of MGITC to increase the colloidal stability, and both colloids were centrifuged twice 1000 *g* × 30 min. The pellets were resuspended in the same initial volume of borate buffer at 10 mM pH = 8.0.

#### Conjugation of SERS-Encoded NPs with Histamine and Parvalbumin Antibodies

For the antibody conjugation, 1 μL (1 mg/mL in PBS 1×) of histamine antibody was added to 750 μL of malachite green SERS tag, and 2 μL (0.38 mg/mL in PBS 1×) of parvalbumin antibody was added to 750 μL of rhodamine B SERS tag in borate buffer 10 mM and pH = 8.5. The colloids were mixed with a vortex and kept undisturbed at room temperature for 90 min. To block the remaining free surface of the NPs, 100 μL of BSA (1 mg/mL in borate buffer) was added, and the mixture was incubated for 30 min. After incubation steps, two centrifugations at 1000 *g* × 30 min were done. The first centrifugation pellet was redispersed in 750 μL of borate buffer and, the second one in 50 μL of a BSA – Sucrose (1% - 10% w/w respectively in phosphate buffer 10 mM pH 7.4). It should be noted that borate buffer at pH 8.4 allowed a better bioconjugation of the nanoparticles, while phosphate buffer at pH 7.4 was chosen for the running on the basis of a better LFIA performance.

Note: The antibody antiparvalbumin shows less binding affinity toward the parvalbumin than the antihistamine toward the histamine, the malachite green SERS tags (resonant with the Raman excitation laser line) were functionalized with the antibody antiparvalbumin.

#### Parvalbumin Protein Extraction

The parvalbumin extraction was performed following an extraction protocol reported by Carrera et al.^63^ Sarcoplasmic protein extraction was carried out by homogenizing 5 g of white muscle in 10 mL of 10 mM Tris–HCl pH 7.2, supplemented with 5 mM PMFS, for 30 s in an Ultra-Turrax device (IKA-Werke, Staufen, Germany). The sarcoplasmic protein extracts were then centrifuged at 40,000 *g* for 20 min at 4 °C (J221-M centrifuge; Beckman, Palo Alto, CA). Parvalbumins were purified by taking advantage of their thermostability, heating the sarcoplasmic extracts at 70 °C for 5 min. After centrifugation at 40,000 *g* for 20 min (J221-M centrifuge, Beckman, Palo Alto, CA), supernatants composed mainly of parvalbumins were quantified by the bicinchoninic acid (BCA) method (Sigma-Chemical Co., USA).

#### LFIA Strip Fabrication

To fabricate the strip, the nitrocellulose membrane was attached to a plastic backing card. The control line of the strips was prepared by dispensing 1 mg/mL of protein G. For the two test line immunosensors, the test lines were prepared by dispensing 0.5 mg/mL of histamine-BSA antigen and 2.5 mg/mL of parvalbumin. The established order of the lines was: control line (line above), parvalbumin test line (line in the middle), and histamine test line (line below). For the one test line immunosensor, a mixture of 0.5 mg/mL histamine-BSA antigen and 2.5 mg/mL parvalbumin were dispensed. All of the lines were dispensed with the IsoFlow dispenser onto a nitrocellulose membrane at a dispensing ratio of 0.100 μL/mm. The strips were dried at 37 °C for 30 min. The absorbent pad was attached to the end of the membrane on the backing card with an overlap between them of around 2.5 mm. The complete strip was cut into individual 5 mm strips.

#### Histamine and Parvalbumin Calibration Curve Procedure

Different concentrations of histamine (2.5–5 × 10^–6^ mg/mL) and parvalbumin (0.5–2.5 × 10^–4^ mg/mL) solutions were prepared in PBS 1×. For the calibration curves, in a 96-well assay plate were mixed 10 μL of histamine or parvalbumin of different concentrations, 10 μL of PBS 1×, 4 μL of each SERS tag, and 80 μL of running buffer (1% casein (w/w), 3% Tween 20 (w/w) in phosphate buffer 10 mM and pH 7.4), and a strip is introduced in the mixture. After 20 min, 20 μL of running buffer was added to clean the strips.

#### Canned Tuna Fish Sample Preparation and Test in the Two-Test-Line Sensor

Several cans of tuna were obtained from a local supermarket. The tuna was dried using absorbent paper. Two grams of the dry tuna was mixed with 8 mL of PBS 1× pH 7.4. The mixture was stirred overnight. The supernatant was filtered with a 0.22 μm filter and diluted 10 times with 1× PBS at pH = 7.4. Later, different concentrations of histamine (0.04, 0.4, 2, 4, 10, 20, 30, 40, 100, 300, and 4000 mg/kg) and/or parvalbumin (4, 10, 20, 30, 40, 100, 200, 300, 400, 2000, and 4000 mg/kg) were spiked in the sample. Then, in a 96-well assay plate were mixed 20 μL of the sample, 4 μL of each SERS tag, and 80 μL of running buffer (1% casein (w/w), 3% Tween 20 (w/w) in phosphate buffer 10 mM and pH 7.4), and a strip is introduced in the mixture. After 20 min, 20 μL of running buffer was added to clean the strips. It should be noted that before the addition of histamine and parvalbumin, the different extracts were analyzed with the LFIA test, showing in all the cases the absence of both allergens.

#### Canned Tuna Fish Sample Test in the Single-Test-Line Sensor

Different extracts of the tuna canned sample were spiked with parvalbumin and histamine to reach a final concentration of 1.25 and 0.5 mg mL^–1^ (equivalent to 50 g/kg Parvalbumin and 20 g/kg histamine), respectively. Then, in a 96-well assay plate were mixed 20 μL of the sample (with histamine, parvalbumin, or blank), 4 μL of each SERS tag, and 80 μL of running buffer (1% casein (w/w), 3% Tween 20 (w/w) in phosphate buffer 10 mM and pH 7.4), and a strip is introduced in the mixture. After 20 min, 20 μL of running buffer was added to clean the strips.

## Data Availability

The data that support the findings of this study are available at ZENODO, doi:10.5281/zenodo.10036362.

## Supporting Information Available

SERS spectra of the SERS tags and their assignments; optimization of running buffers and pHs; calibration curves; and fittings for parvalbumin and histamine (PDF)
