# Supplementary material for: Au@Ag Core–Shell Nanoparticles for Colorimetric and Surface-Enhanced Raman-Scattering-Based Multiplex Competitive Lateral Flow Immunoassay for the Simultaneous Detection of Histamine and Parvalbumin in Fish
Source: ACS Appl Nano Mater. 2023 Dec 19;7(1):498–508. doi: 10.1021/acsanm.3c04696 (PMC10788866; doi:10.1021/acsanm.3c04696)
Supplement: Supplementary file 1 — an3c04696_si_001.pdf [file an3c04696_si_001.pdf]

Supporting Information

for

Au@Ag Core-Shell Nanoparticles for Colorimetric and SERS-Based Multiplex Competitive Lateral Flow Immunoassay for the Simultaneous Detection of Histamine and Parvalbumin in Fish

Carlos Fernández-Lodeiro,[1,2,3†] Lara González-Cabaleiro,[1,2,3†] Lorena Vázquez-Iglesias,[1,2,3] Esther Serrano-Pertierra,[4] Gustavo Bodelón,[1,5] Mónica Carrera,[6] María Carmen Blanco-López,[7] Jorge Pérez-Juste[1,2,3*] and Isabel Pastoriza-Santos[1,2,3*]

[1]CINBIO, Universidade de Vigo, Campus Universitario As Lagoas, Marcosende, 36310 Vigo, Spain

[2]Department of Physical Chemistry, Universidade de Vigo, Campus Universitario As Lagoas, Marcosende, 36310 Vigo, Spain

[3]Galicia Sur Health Research Institute (IIS Galicia Sur), 36310 Vigo, Spain

[4]Department of Biochemistry and Molecular Biology & Institute of Biotechnology of Asturias, University of Oviedo, 33006, Oviedo, Spain

[5]Department of Functional Biology and Health Sciences, Universidade de Vigo, 36310 Vigo, Spain

[6]Department of Food Technology, Spanish National Research Council, Marine Research Institute, 36208 Vigo, Spain

[7]Department of Physical and Analytical Chemistry & Institute of Biotechnology of Asturias, University of Oviedo, c/Julián Clavería 8, 33006, Oviedo, Spain

[†] Equal contribution

*Corresponding authors

Jorge Pérez-Juste mail: juste@uvigo.gal

and Isabel Pastoriza-Santos: pastoriza@uvigo.gal

A

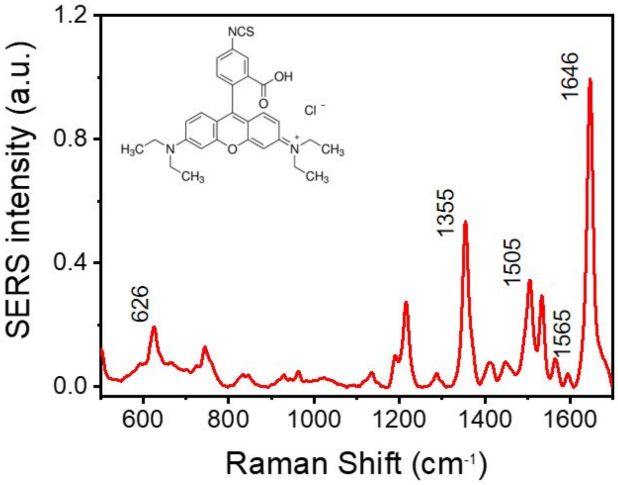

B

SERS peak position (cm^{-1})	Peak assignment
1646	C_X–C_X str
1565	C_X–C_X str
1505	C_X–C_X str
1355	C_X–C_X str
626	C_X–C_X-C_X bend

Figure S1. (A) SERS spectrum of RBITC SERS tag. **(B) S**elected SERS peaks of RBITC with their vibrational assignment.[1] Abbreviations: str = stretching, X = xanthene ring.

A

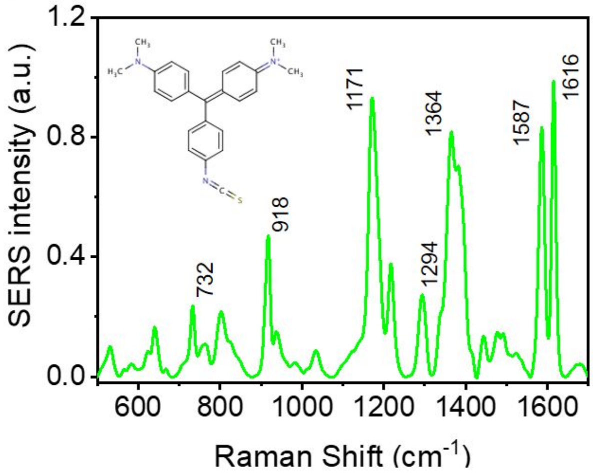

B

SERS peak position (cm^{-1})	Peak assignment
1616	N-Ph ring + C-C str
1587	In plane ring, str + bend
1364	N-Ph ring str
1294	In plane C-H and C-C-H
1171	ν_9 benzene in plane
918	b_{1u} in plane benzene ring
732	In-plane benzene ring + NH_2 str

Figure S2. (A) SERS spectrum of MGITC SERS tag. **(B) S**elected SERS peaks of RBITC with their vibrational assignment.[2] Abbreviations: str = stretching.

.

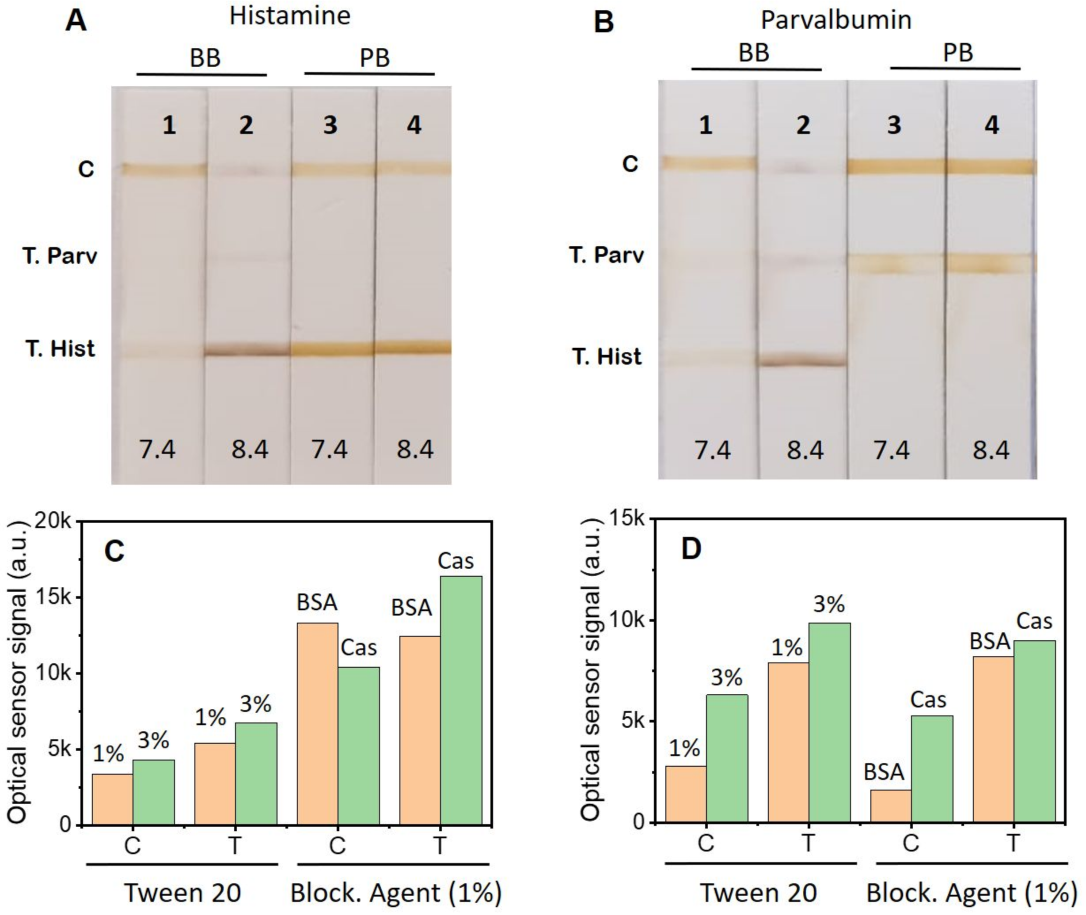

Figure S3. Optimization of the running buffer. (A-B) Photographs of LFIA strips for histamine (A) and parvalbumin (B) detection in borate buffer (BB) and phosphate buffer (PB) at the indicated pHs. (C) Quantification of colorimetric signal intensity in test (T) and control (C) lines in LFIA strips for histamine detection in PB at pH 7.4 and varying the Tween® 20 (1% and 3%) concentration or BSA and casein (Cas) as blocking agent (Block. Agent) in the running buffer. (D) Quantification of colorimetric signal intensity in test (T) and control (C) lines in LFIA strips for parvalbumin detection varying the Tween® 20 concentration (surfactant, surf1% and 3%) or BSA and casein as blocking agent in the running buffer.

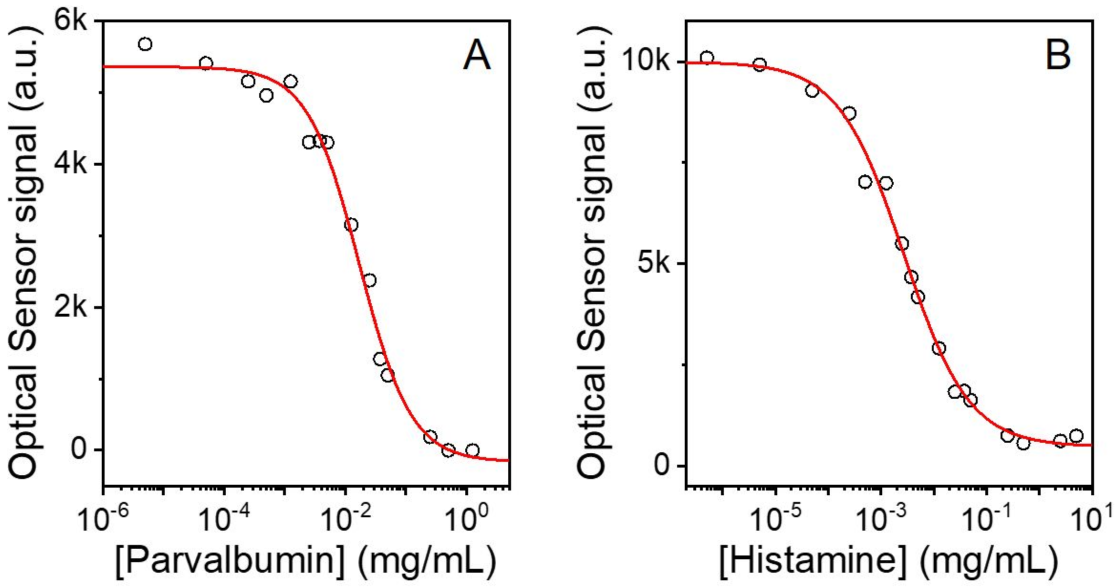

Figure S4. (A-B) Quantification of LFIAs for parvalbumin (A) and histamine (B) shown in Figure 3A and 3B by the optical reader in PB. The red lines represent the fitting of the data to a four-parameter logistic (4PL) equation. The table S1 summarizes the fitting values.

Table S1. Four-parameter logistic equation values from the calibration curves of parvalbumin and histamine using the colorimetric LFIA method in PB.

Antigen	A_1	A_2	X_0 (mg·mL^{-1})	P	R^2	IC_{10}/LOD (mg·mL^{-1})	IC_{20} (mg·mL^{-1})	IC_{80} (mg·mL^{-1})
Parvalbumin	5355.7 ± 128.4	-161.6 ± 181.4	0.01 ± 0.002	1.02 ± 0.12	0.98	1.96×10^{-3}	0.004	0.06
Histamine	9987.0 ± 200.1	481.6 ± 175.5	$(2.70 \pm 0.32)\times10^{-3}$	0.70 ± 0.05	0.99	1.18×10^{-4}	3.75×10^{-4}	0.02

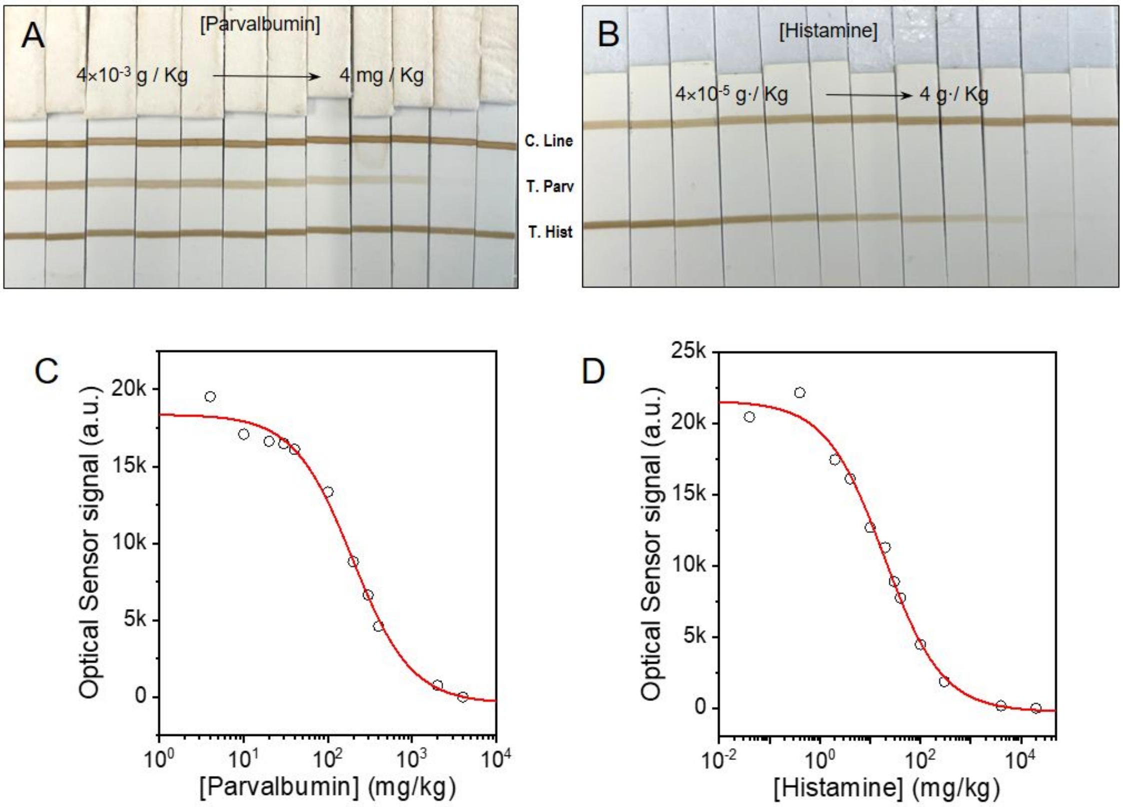

Figure S5. (A and B) Photographs of LFIA strips from extract samples diluted in PBS and containing different concentrations of parvalbumin (A) and histamine (B). It should be noted that just the T line for histamine is present in B. (C and D) Quantification of LFIAs for parvalbumin (A) and histamine (B) in canned tuna extract by the optical reader. The red lines represent the fitting of the data to a 4PL equation. The Table S2 summarizes the fitting values.

Table S2. Four-parameter logistic equation values from the calibration curves of parvalbumin and histamine using the colorimetric LFIA method in canned tuna extract.

Antigen	A_1	A_2	X_0 (mg·kg^{-1})	P	R^2	IC_{10}/LOD (mg·kg^{-1})	IC_{20} (mg kg^{-1})	IC_{80} (mg kg^{-1})
Parvalbumin	18380.0 ± 586.2	-416.4 ± 726.9	195.7 ± 21.0	1.24 ± 0.18	0.98	33.4	94.1	597.5
Histamine	21593.9 ± 767.5	-255.1 ± 643.5	18.9 ± 3.1	0.75 ± 0.09	0.98	1.0	3.0	120.5

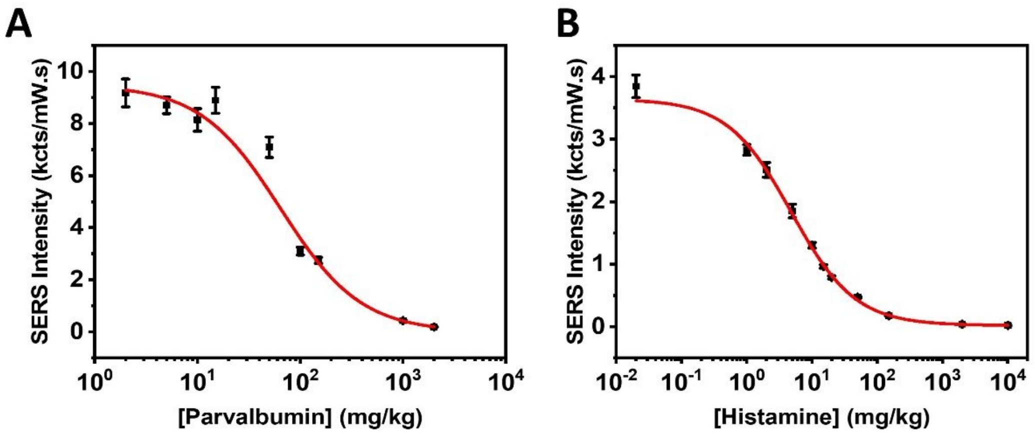

Figure S6. (A and B) Variation of SERS intensity at 1646 cm^{-1} (A) or 1616 cm^{-1} (B) with the concentration of parvalbumin and histamine, respectively, extracts of canned tuna. The red lines represent the fitting of the SERS intensity measurements to a four-parameter sigmoid equation. Standard deviations correspond to the 20 higher-intensity SERS points of each strip. All SERS measurements were carried out with a 532 nm laser line, 10x objective, 0.25, 2.31 or 12.50 mW laser power depending on the color intensity of the test lines, 1.0 s acquisition time and 143 points.

Table S3. Four-parameter logistic equation values from the calibration curves of parvalbumin and histamine using the colorimetric LFIA method in canned tuna extract.

Antigen	A_1	A_2	X_0 ($mg\cdot kg^{-1}$)	P	R^2	IC_{10}/LOD ($mg\cdot kg^{-1}$)	IC_{20} ($mg\ kg^{-1}$)	IC_{80} ($mg\ kg^{-1}$)
Parvalbumin	9479.2 ± 833.3	-11.4 ± 118.4	64.3 ± 14.6	1.1 ± 0.2	0.99	8.9	18.4	224.5
Histamine	3641.7 ± 254.7	18.2 ± 4.1	4.8 ± 0.8	0.89 ± 0.04	0.99	0.4	1.0	22.9

References

(1) Hildebrandt, P.; Stockburger, M. Surface-Enhanced Resonance Raman Spectroscopy of Rhodamine 6G Adsorbed on Colloidal Silver. *J. Phys. Chem.* **1984**, *88* (24), 5935–5944.

(2) Lueck, H. B.; Daniel, D. C.; McHale, J. L. Resonance Raman Study of Solvent Effects on a Series of Triarylmethane Dyes. *J. Raman Spectrosc.* **1993**, *24* (6), 363–370.
